# Frequency of iron deficiency anemia (IDA) among patients with Helicobacter pylori infection

**DOI:** 10.12669/pjms.37.3.3944

**Published:** 2021

**Authors:** Adeel Rahat, Lubna Kamani

**Affiliations:** 1 Dr Adeel Rahat, FCPS. Instructor, Gastroenterology Section, Aga Khan University Hospital, Karachi, Pakistan; 2 Dr Lubna Kamani FCPS, MRCP (UK), FRCP(London), FACG Associate Professor and Director GI residency program Gastroenterology Department, Liaquat National Hospital, Karachi, Pakistan

**Keywords:** Helicobacter pylori infection, iron deficiency anemia, anemia

## Abstract

**Background and Objective:**

Helicobacter Pylori (H. pylori) is a widespread infection across the globe having a high prevalence among the developing countries. Iron Deficiency is anticipated to be the most prevalent micronutrient deficiency globally, the most frequent cause of anemia. Our objective was to determine frequency of Iron Deficiency Anemia (IDA) among patients with H. Pylori gastritis.

**Methods:**

It was a cross-sectional prospective study. Patients fulfilling inclusion criteria were enrolled at Liaquat National Hospital, Karachi, Pakistan. Blood samples were taken for serum iron, transferrin saturation, ferritin, and total iron-binding capacity and H.pylori assessed by urea breath test, stool for antigen, Rapid urease test or histopathology.

**Results:**

112 patients with H. Pylori infection with anemia were included. 53 (47.3%) were males & 59 (52.7%) were females with mean age of 38.4464 ± 9.00634 years. Iron deficiency anemia was seen in 42 patients (37.5%).

**Conclusion:**

IDA was noted in 37.5% of cases. H. Pylori infection is a frequent cause of iron-deficiency anemia of previously unidentified origin among adults.

## INTRODUCTION

*H. Pylori* is a chronic microbial infection, which is highly prevalent around the globe, especially in developing countries. The worldwide prevalence of *H. Pylori* is recorded to be about 50%. Though high variation has been associated with age, geography, and socioeconomic status, its overall prevalence is high in developing countries due to many reasons.^1^^.^

*H. Pylori* infection affects people from all across the globe but its prevalence differs from one region to the other.^1^ Usually acquired in childhood in the early stages, it can become chronic if untreated.^2^ The people who acquire this infection mostly do not show many symptoms, which leads to the hypothesis that some of *H. Pylori* strains are not harmful or even beneficial^3^ and may lead to illness in a very small number of adults.^4^ It can be a causative factor for multiple upper gastrointestinal diseases like gastritis, gastric, or duodenal ulceration, and it even augments the risk for gastric malignancy.^5^ As per the study conducted by Ford AC et al about the epidemiological aspects of *H. Pylori*, and the implications it has on public health; the important risk factors proposed for infection include growing age, shorter height, male sex, obesity, tobacco usage, poor socioeconomic conditions and low educational standing of the parents in studies conducted among children.^6^ Multiple diagnostic modalities are available with varying sensitivity and specificity for assessing *H. Pylori* infection. These include serology, urea breath test (UBT), Rapid Urease Test (RUT), biopsy with histopathology, and cultures. The most specific way remains the isolation of the microbe from gastric biopsies to establish the diagnosis of infection. Rasool et al conducted a study in 2007 which showed that *H. Pylori* was diagnosed by rapid urease test and histology in 61 (65%) and 66 (70%) patients respectively, while 14C UBT helped diagnosing infection in 63 (67%) patients. UBT’s accuracy was found to be 93% in comparison with histology with a high positive predictive value of 97% and the negative predictive value was 84%.^7^

Anemia, described as a reduction in the quantity of red blood cells (RBCs) or the quantity of hemoglobin (Hb) concentration below established cut-off levels, is an international public health issue. According to the World Health Organization Database on Anemia (1993-2005), almost a quarter of the world’s population is anemic.^8^ *H. pylori* infection (active state) was independently related to iron deficiency and the resultant anemia^9^ and there are also studies showing a poor response of anemia to oral iron replacement with coexistent active *H. Pylori* infection.^10^ Valiyaveetil et al conducted a randomized control study in 2004 that suggested that treatment of *H. Pylori* infection may lead to enhancement of response to oral iron therapy^10^ . Eradicating *H. Pylori* results in an enhanced response to oral iron replacement among infected pregnant female patients having Iron deficiency anemia.^11^

This study evaluated the frequency of IDA among anemic patients with *H. Pylori* infection. Multiple studies point toward a positive linkage between *H. Pylori* infection and anemia secondary to iron deficiency.^12^-^14^ However, the evidence is still insufficient in a Pakistani population. The results of this study will aid the clinicians in identifying patients who are at increased risk of developing anemia secondary to iron deficiency. Its early detection and proper management will, hence, save the patients from anemic heart failure, which happens to be a complication for chronic anemia. This will also upgrade the lifestyle of patients by improving the signs and symptoms of anemia like lethargy and easy fatigability.

## METHODS

Adopting the cross-sectional approach, this study was conducted after hospital ethics committee approval (Ref: App#0486-2019-LNH-ERC, Dated: June 3, 2019) at Liaquat National Hospital, Karachi in the Department of Gastroenterology, from July 29^th^, 2019 till Jan 28^th^, 2020. Patients that were enrolled were the ones attending the in-patient or out-patient facilities at the Gastroenterology Department at Liaquat National Hospital, Karachi with presence of *H.pylori* antigen in stool test or positive urea breath test or chronic gastritis because of *H. Pylori* on endoscopy & gastric biopsy with anemia. For all patients included in this study, the following information were collected: age, gender, nutritional history and menstrual history in female patients. Patients were excluded if they had any other source of chronic blood loss. Blood samples were collected for calculating serum iron and ferritin concentrations, transferrin saturation, and total iron-binding capacity (TIBC). Patients were labeled having Iron Deficiency Anemia when the concentration of hemoglobin was less than 12 g/dl in males and less than11 g/dL in females, and further serum studies showed a ferritin level of < 30 ng/ ml with a raised Total Iron Binding Capacity greater than 450 μg/dL, Serum Iron Level less than 50 μg/dL, reduced transferrin saturation less than 20%. Clinical history along with demographics were recorded by a principal investigator as per the predesigned pro forma, and a documented informed consent was attained ahead of enrolling the patient for the study. To avoid confounding variables, strict adherence was done to the inclusion and exclusion criteria.

### Statistical analysis:

For data analysis, SPSS version 22 was utilized. Percentages and frequencies were recorded for categorical variables like gender, education level, socioeconomic status, hemoglobin levels, and other parameters like serum Iron, Ferritin, transferrin saturation, and TIBC levels, iron deficiency anemia. Values were calculated as mean ± standard deviation for continuous variables such as age. Effect modifiers like age, gender, education level, socioeconomic status, Hb level were addressed via stratification. Chi-square test was applied. P ≤ 0.05 was considered as level of significance.

## RESULTS

Total of 112 patients infected with *H. Pylori* with anemia were registered for this study. The mean age of 38.4464 ± 9.00634 years was observed. Age distribution is shown in Graph-1. The descriptive statistics in relation to age is shown in Table-I.

**Graph-I:**
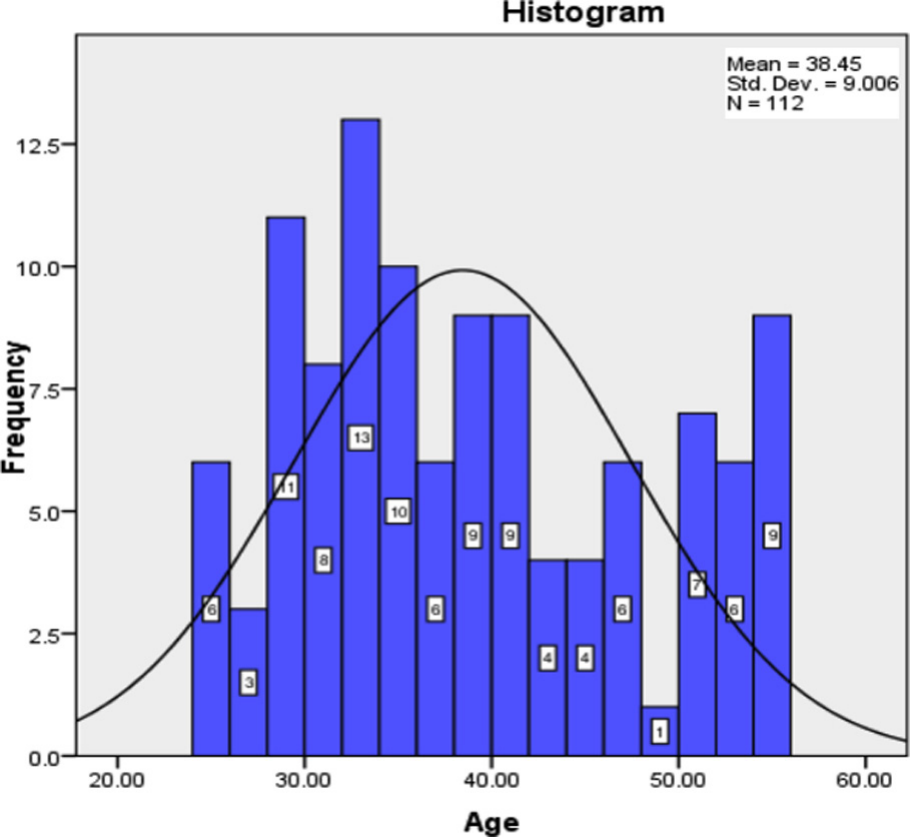
Frequency distribution of Age (years).

**Table-I:** Descriptive statistics of age, Socioeconomic status, Hemoglobin level, Serum iron level, Serum ferritin level, Total iron-binding capacity & Transferrin saturation.

VARIABLE	MEAN±SD
Age (years)	38.4464 ± 9.00634
Socioeconomic status (rupees)	42366.0714 ± 23660.890
Hemoglobin level (g/dl)	10.2188 ± 0.76164
Serum iron level (ug/dl)	52.633 ± 15.289
Serum ferritin level (ng per ml)	125.8929 ± 78.69777
Total iron binding capacity (ug/dl)	285.4911 ± 150.78916
Transferrin saturation (%)	25.9603 ± 14.71376

The mean hemoglobin level was 10.2188 ± 0.76164 g/dl. The mean serum iron level was 52.633 ± 15.289 ug/dl.. The mean serum ferritin level was 125.8929 ± 78.69777 ng per ml. The mean total iron-binding capacity was 285.4911 ± 150.78916 ug/dl.

The mean transferrin saturation was 25.9603 ± 14.71376%. The descriptive statistics of socioeconomic status, Hemoglobin levels, Serum Iron, Serum Ferritin, TIBC, and transferrin saturation are shown in Table-I.

Fifty three patients (47.3%) were males & 59 patients (52.7%) were females (as shown in Table-II). In this study education level was matriculation in 47 patients (42%), intermediate in 35 (31.3%), and graduation in 30 (26.8%).Socioeconomic status/monthly income was < 10000 in 2 patients (1.8%), 10000-25000 in 22 (19.64%) and > 25000 in 88 (78.57%). Iron deficiency anemia was seen in 42 patients (37.5%).

**Table-II:** Frequency distribution of gender, education level, socioeconomic status, H.pylori detection method, iron deficiency anemia (n=112).

Gender	Frequency (n)	Percentage (%)
Male	53	47.3%
Female	59	52.7%
Total	112	100%

*Education level*	*Frequency (n)*	*Percentage (%)*

Matriculation	47	42%
Intermediate	35	31.3%
Graduation	30	26.8%
Total	112	100%

*Socioeconomic status (rupees)*	*Frequency (n)*	*Percentage (%)*

< 10000	2	1.8%
10000-25000	22	19.64%
> 25000	88	78.57%
Total	112	100%

*H.pylori detection method*	*Frequency (n)*	*Percentage (%)*

Stool for H.pylori Antigen	22	19.6%
Urea Breath Test	28	25%
Rapid Urease Test	9	8%
Histopathology	53	47.3%

*Iron deficiency anemia*	*Frequency (n)*	*Percentage (%)*

Yes	42	37.5%
No	70	62.5%

The frequencies of age groups, gender, education level, and socioeconomic status were calculated according to iron deficiency anemia. The results are shown in Table-III. In this study, anemia secondary to iron deficiency was significantly associated with age (p-value=0.042), while no association was observed with gender, educational level & socioeconomic status with the p-value of 0.732, 0.813 & 0.068 respectively.

**Table-III:** Iron deficiency anemia according to Age, Gender, Education level & Socioeconomic status.

Age (years)	Iron deficiency anemia		Total	P-Value

	Yes	No		
25-40 Years	22(19.64%)	50(44.64%)	72(64.3%)	0.042
41-55 Years	20(17.85%)	20(17.85%)	40(35.7%)	
Total	42(37.5%)	70(62.5%)	112(100%)	

*Gender*	*Iron deficiency anemia*		*Total*	*P-Value*

	*Yes*	*No*		

Male	19(17%)	34(30.4%)	53(47.32%)	0.732
Female	23(20.5%)	36(32.1%)	59(52.67%)	
Total	42(37.5%)	70(62.5%)	112(100%)	

*Education level*	*Iron deficiency anemia*		*Total*	*P-Value*

	*Yes*	*No*		

Matriculation	16(14.3%)	31(27.7%)	47(41.96%)	0.813
Intermediate	14(12.5%)	21(18.8%)	35(31.25%)	
Graduation	12(10.7%)	18(16.6%)	30(26.78%)	
Total	42(37.5%)	70(62.5%)	112(100%)

*Socioeconomic status*	*Iron deficiency anemia*		*Total*	*P-Value*

	*Yes*	*No*		

< 10000	1(0.9%)	1(0.9%)	2(1.8%)	0.068
10000-25000	6(5.35%)	16(14.28%)	22(19.64%)	
> 25000	35(31.25%)	53(47.32%)	88(78.57%)	
Total	42(37.5%)	70(62.5%)	112(100%)	

## DISCUSSION

In this study, iron deficiency anemia was noted in 42 patients (37.5%) with *H. Pylori* infection, as compared to results of the Monzón et al^14^ study, which had stated that 38% of the patients may have iron deficiency anemia due to *H. pylori* infection, it also suggests that *H. pylori* gastritis can be a common etiological reason for IDA among adult patients with iron deficiency/iron refractoriness among whom the routine work-up for diagnosing the cause of IDA yielded no obvious result. One previous study stated that a large proportion of patients having atrophic body gastritis also encounter IDA and out of these, 61 % were diagnosed with *H. Pylori* infection.^15^ A Korean study on adolescents (n=937) showed positive seropositivity rate for *H. Pylori* with iron deficiency to be 35.3%.^16^

In Monzón et al study 14, eradication of *H. pylori* was linked with resolution of IDA without any additional iron replacement therapies and a relapse-free period of approximately 24 months mean follow-up. These results support in favor of the association of *H. pylori* infection with iron deficiency anemia. The Objective Response (OR) of infection with *H. pylori* as the causative reason for IDA was as high as ten times in the second group as compared to the first one.

In this study IDA was noted in 17% male patients and 20.5% female patients as compared to an earlier study that reported Iron Deficiency Anemia’s prevalence among dyspeptic patients to be 26.9%, 35.2% in men, and 64.8% in women. Anemia’s prevalence among patients with *H. Pylori* gastritis was 30.9% and 22.5% among those who were not infected^12^. Thus, a hypothesis was put forth that *H. Pylori* -association with anemia was a result of reduced iron absorption in the context of hypochlorhydria^13^

The mean hemoglobin level in this study was 11.830 ± 1.695 g/dl and the mean transferrin saturation was 27.693 ± 12.695%. Patients having both, *H. Pylori* gastritis and Iron Deficiency Anemia are more prone to have corpus gastritis than those who have *H. Pylori* -infection but not anemia.^15^ Because of corpus gastritis, reduced gastric acid secretion and raised intragastric pH may ensue which results in impairment of iron absorption.^15^ However, gastric acid secretion may normalize after eradicating *H. Pylori*. Likewise, another significant consequence of *H. Pylori* gastritis that results in decreased absorption of iron is a decrease in gastric juice ascorbic acid concentration as ascorbic acid aids in iron absorption from the gut by its reduction into the ferrous form.^17^

Another method that has been hypothesized to understand the relation between iron deficiency and *H. Pylori* gastritis was iron uptake by the bacterium itself. Various microorganisms use iron as a growth factor and *H. Pylori* is one of them. It contains a 19-kDa iron-binding protein resembling ferritin and thus may play a pivotal role in storing excess iron by the *H. Pylori* 18

There is another possible mechanism that explains the reduced availability of iron which is seizing up of iron because of lactoferrin in the gastric mucosa. *H. Pylori* sequesters iron from human lactoferrin through a receptor-mediated mechanism^19^^.^ It appears that the gastric mucosal lactoferrin secretion is affected by the *H. Pylori* 20 . Lactoferrin levels of the gastric wall are reported to be considerably higher in *H. Pylori* positive IDA patients than the persons who were not anemic and also negative for *H. Pylori*, non-anemic but positive for *H. Pylori*, and *H. Pylori* negative with IDA. This shows that lactoferrin possibly plays an important role in iron deficiency anemia.^16^

In this study, 52.7% of patients were females and IDA was predominant in the female gender. Results of the study of Monzón et al^14^ on premenopausal women disagree with earlier results of Annibale et al^15^. The reason was that they showed that 92% of the patients, mainly premenopausal females, recovered from anemia at one year of follow-up after *H. Pylori* eradication. The discrepancies have more to do with the definition of response.

There may be certain other factors that are responsible for iron deficiency anemia in otherwise healthy normal premenopausal females. These mainly include increased blood loss during menstrual flow, pregnancy induced higher iron demands, dietary insufficiency, and breast-feeding.^21^ Menstrual blood loss may be reduced by approximately 50% by hormonal contraceptive therapy. This may help in females with average or mildly above-average blood loss^22^. Monzón et al^14^ study showed that this therapy was also helpful in resolving IDA in those premenopausal females in whom the requirements of iron were increased despite of eradication of *H. pylori*.

*H. pylori* infection may also result in Latent Deficiency, which may improve after the infection has been irradicated^23^,^24^ . However, it is not known if *H. Pylori* -infected patients who simultaneously have Latent Deficiency are at higher risk of having IDA or not.

In conclusion, the results of this current study show that *H. pylori* infection is a common cause of IDA among females and patients with lower education levels.

### Limitation of the Study:

The main limitations were relatively smaller sample size, and improvement in anemia following *H. pylori* eradication. So additional studies with larger sample sizes are suggested.

## CONCLUSION

Iron Deficiency Anemia (IDA) was noted in 37.5% of cases. *H. pylori* infestation is a frequent cause of iron-deficiency anemia of previously unknown origin among adults.
